# Novel PET Imaging of Inflammatory Targets and Cells for the Diagnosis and Monitoring of Giant Cell Arteritis and Polymyalgia Rheumatica

**DOI:** 10.3389/fmed.2022.902155

**Published:** 2022-06-06

**Authors:** Kornelis S. M. van der Geest, Maria Sandovici, Pieter H. Nienhuis, Riemer H. J. A. Slart, Peter Heeringa, Elisabeth Brouwer, William F. Jiemy

**Affiliations:** ^1^Department of Rheumatology and Clinical Immunology, University of Groningen, University Medical Center Groningen, Groningen, Netherlands; ^2^Department of Nuclear Medicine and Molecular Imaging, Medical Imaging Center, University Medical Center Groningen, University of Groningen, Groningen, Netherlands; ^3^Department of Biomedical Photonic Imaging Group, University of Twente, Enschede, Netherlands; ^4^Department of Pathology and Medical Biology, University of Groningen, University Medical Center Groningen, Groningen, Netherlands

**Keywords:** PET/CT, giant cell arteritis (GCA), polymyalgia rheumatica (PMR), radiotracer, imaging, large-vessel vasculitis (LVV)

## Abstract

Giant cell arteritis (GCA) and polymyalgia rheumatica (PMR) are two interrelated inflammatory diseases affecting patients above 50 years of age. Patients with GCA suffer from granulomatous inflammation of medium- to large-sized arteries. This inflammation can lead to severe ischemic complications (e.g., irreversible vision loss and stroke) and aneurysm-related complications (such as aortic dissection). On the other hand, patients suffering from PMR present with proximal stiffness and pain due to inflammation of the shoulder and pelvic girdles. PMR is observed in 40–60% of patients with GCA, while up to 21% of patients suffering from PMR are also affected by GCA. Due to the risk of ischemic complications, GCA has to be promptly treated upon clinical suspicion. The treatment of both GCA and PMR still heavily relies on glucocorticoids (GCs), although novel targeted therapies are emerging. Imaging has a central position in the diagnosis of GCA and PMR. While [^18^F]fluorodeoxyglucose (FDG)-positron emission tomography (PET) has proven to be a valuable tool for diagnosis of GCA and PMR, it possesses major drawbacks such as unspecific uptake in cells with high glucose metabolism, high background activity in several non-target organs and a decrease of diagnostic accuracy already after a short course of GC treatment. In recent years, our understanding of the immunopathogenesis of GCA and, to some extent, PMR has advanced. In this review, we summarize the current knowledge on the cellular heterogeneity in the immunopathology of GCA/PMR and discuss how recent advances in specific tissue infiltrating leukocyte and stromal cell profiles may be exploited as a source of novel targets for imaging. Finally, we discuss prospective novel PET radiotracers that may be useful for the diagnosis and treatment monitoring in GCA and PMR.

## Introduction

Giant cell arteritis (GCA) and polymyalgia rheumatica (PMR) are two related inflammatory diseases exclusively affecting adults above the age of 50, with a peak incidence between 75 and 79 years of age (1). GCA is a vasculitis affecting medium- to large-sized arteries which can be subclassified into a spectrum that includes cranial GCA (C-GCA) and large-vessel GCA (LV-GCA) (2). C-GCA mainly affects the cranial arteries and leads to ischemic symptoms such as jaw claudication, vision loss, and stroke (3, 4). LV-GCA mainly affects the aorta and its main branches and may lead to aneurysm formation and aortic dissection. Up to 83% of GCA patients present with overlapping C-GCA and LV-GCA (5). PMR is a rheumatic inflammatory disorder characterized by inflammation of bursae, tendon sheaths, and joints primarily affecting the shoulder and pelvic girdles (6). GCA and PMR commonly coexist; up to 60% of GCA patients are diagnosed with PMR while up to 21% of PMR patients present with overlapping GCA (7). To date, glucocorticoid (GC) therapy remains the mainstream treatment for the management of GCA and PMR (8). Although GC treatment is effective in inducing and maintaining remission, it can cause substantial toxicity in patients (9). Recently, IL-6 receptor blocking therapy has shown positive results as GC sparing therapy in GCA (10). Promising results with this therapy have also been reported in PMR (11, 12). However, tocilizumab monotherapy is not recommended for these diseases and combination treatment with GC is still imperative, especially in GCA.

Historically, the diagnosis of GCA solely relied on the assessment of clinical signs and symptoms, laboratory assessment of inflammatory markers such as elevated C-reactive protein (CRP) and erythrocyte sedimentation rate (ESR), and positive histological evidence of giant cell arteritis in the temporal artery biopsy (TAB) (13). Similarly, diagnosis of PMR also relies heavily on the assessment of clinical signs and symptoms, and laboratory assessment of inflammatory markers (14). More recently, imaging techniques such as ultrasonography and [^18^F]fluorodeoxyglucose (FDG)-positron emission tomography (PET) have gained importance as diagnostic tools for GCA and PMR, whereas these imaging techniques are also increasingly used for treatment monitoring (11, 15–18). Previously, [^18^F]FDG-PET/CT was only utilized for the detection of LV-GCA due to its limitation in resolution. However, [^18^F]FDG-PET/CT employing new generation scanners with improved resolution has been shown to be able to detect C-GCA. Recent reports have shown that C-GCA can be effectively detected by PET/CT (up to 83% sensitivity and 100% specificity) (19, 20). However, despite its utility, there are several important clinical drawbacks posed by [^18^F]FDG-PET as a diagnostic tool in the diagnosis of GCA and PMR. Firstly, [^18^F]FDG uptake is non-specific and only indicates increased glucose metabolism. Therefore, it may be present in the context of neoplasia, inflammation, degenerative disease, and increased muscle use (21). In the context of vascular inflammation, [^18^F]FDG may also be taken up due to atherosclerotic activity (21). In addition, [^18^F]FDG shows intense uptake in several organs that may hamper its diagnostic accuracy. One example is the high brain uptake of [^18^F]FDG that may result in a low target-to-background ratio (TBR) in cranial vessels and limit the diagnostic accuracy of C-GCA. Furthermore, the diagnostic accuracy of [^18^F]FDG-PET in patients undergoing GC treatment is significantly reduced as exemplified by one study reporting that only 36% of LV-GCA patients showed a positive [^18^F]FDG-PET scan after 10 days of GC treatment (22). A reduction in diagnostic accuracy has also been shown in PMR patients undergoing GC treatment albeit to a lesser extent as compared to GCA (17). GC rapidly blocks glycolysis pathways important for FDG uptake in inflammatory cells (23, 24). It is imperative to start GC treatment upon suspicion of GCA while postponing the GC treatment can be difficult in patients with suspected PMR. Unfortunately, diagnostic imaging of these patients is often not feasible within a narrow timeframe due to limited hospital capacities. Therefore, there is a strong clinical need to identify novel radiotracers that (1) have low background radioactivity in non-target organs and blood pool, and (2) can still accurately detect ongoing inflammation for a prolonged period (e.g., up to weeks) after initiation of GC treatment. Such radiotracers could potentially help to firmly rule in or rule out GCA/PMR and would provide an important benefit to patients in which the diagnosis remains uncertain despite routine clinical evaluation. With the expanding knowledge regarding the cellular heterogeneity at the site of inflammation in GCA and PMR, novel radiotracers targeting these specific cell subsets may prove to be useful for the diagnosis and eventually treatment monitoring in GCA and PMR patients.

## Immunopathology of GCA and PMR

### C-GCA

The immunopathology of GCA is characterized by leukocyte infiltration at the site of inflammation. Although not yet fully understood, decades of efforts in characterizing and understanding the cellular heterogeneity in the inflamed GCA vessels have led us to better understand the pathogenesis of this disease. Based on these data, a pathogenic model has been established in which the initiation of GCA is believed to start with the activation of vascular dendritic cells (vasDCs) through toll-like receptors (TLRs) stimulation by still unknown triggers. Upon activation, these vasDCs adopt a phenotype characterized by the expression of the activation marker CD83 and elevated expression of the costimulatory molecule CD86 (25). These activated vasDCs express the chemokines CCL19 and CCL21 while simultaneously expressing the receptor CCR7, causing these activated vasDCs to be trapped in the vessel wall. Moreover, these activated vasDCs produce CCL20 and a range of proinflammatory cytokines (IL-1β, IL-6, IL-18, IL-23, and IL-33) leading to the recruitment of CD4+ T cells into the vessel-wall, their subsequent co-stimulation and activation, and their polarization into Th1 and Th17 cells (26–29). Although infiltrating T cells in the vessel wall show expression of the inhibitory checkpoint molecule PD-1, vasDCs exhibit low expression of the coinhibitory ligand PD-L1 resulting in dampened negative regulation of T cell activation (30, 31). Activated Th1 and Th17 cells produce high amounts of IFNγ and IL-17, respectively, which in turn activate the resident vascular smooth muscle cells (VSMCs) and endothelial cells (ECs). Activated ECs express high levels of adhesion molecules (VCAM-1, ICAM-1 and E-selectin) enabling leukocyte adhesion and transmigration to the vessel wall (32). Activated VSMCs produce several crucial chemokines such as CXCL9, CXCL10, CXCL11, CXCL13, CCL2, and CX3CL1 augmenting the infiltration of CXCR3+ CD8+ T cells, CXCR3+/CXCR5+ B cells, and CCR2+/CX3CR1+ monocytes to the vessel wall (33–37). Infiltrating CD20+ B cells organize themselves into tertiary lymphoid organs (TLOs) where they produce proinflammatory cytokines which perpetuate the inflammatory processes (38, 39). Infiltrating CD8+ T cells start to produce cytokines such as IFN-γ and IL-17 which triggers a positive feedback loop recruiting more CD4+ and CD8+ T cells as well as monocytes to the vessel wall (36). Notably, activated VSMCs, ECs, CD4+ and CD8+ T cells also produce GM-CSF, a potent hematopoietic growth factor that induces the differentiation and maturation of infiltrating monocytes into proinflammatory CD206+ macrophages (40, 41). These CD206+ macrophages express the collagenase matrix metalloproteinase (MMP)-9 and proangiogenic factor YKL-40 (42). These CD206+/YKL-40+/MMP-9+ macrophages are mainly located in the media and media borders promoting collagen degradation and neovessel formation, enabling more invasion of T cells and monocytes into the vessel wall (42–45). In addition, these CD206+ macrophages express high levels of the growth factor M-CSF, priming adjacent macrophages to become FRβ+ macrophages (41). These M-CSF primed FRβ+ macrophages produce high levels of platelet-derived growth factor (PDGF)-AA which promotes fibroblast migration and proliferation. Furthermore, macrophages are incredibly plastic cells that may change their phenotype in response to cues from the microenvironment. Proinflammatory cytokines such as IFNγ, IL-17, and IL-6 that are abundantly present in the inflamed vessel wall may trigger the expression of a multitude of macrophage markers (5). Notably, abundant numbers of CD64+, CD86+, iNOS+ and CD163+ macrophages have been reported in GCA-affected vessels (41, 46–48). Moreover, these activated macrophages themselves produce a wide range of proinflammatory cytokines (including IL-6, TNF-α, IL-1β, GM-CSF) and growth factors (TGF-β, VEGF, PDGFs) (5). Macrophage secreted proinflammatory cytokines contribute to a positive feedback loop amplifying the inflammatory process. Macrophage secreted growth factors promote fibroblasts and VSMCs activation and differentiation into α-smooth muscle actin (SMA)+ myofibroblasts and subsequently their migration and proliferation in the intima layer which results in intimal hyperplasia and ultimately vessel-wall occlusion (49, 50). Of note, this pathogenic model has largely been constructed from studies on TAB obtained from C-GCA patients.

### LV-GCA

Our understanding of the pathogenesis of LV-GCA is derived from studies with aortic specimens obtained during aortic aneurysm surgery. Aortitis in GCA is characterized by granulomatous inflammation largely occurring in the medial layer of the aorta. This granulomatous inflammation leads to medial necrosis which is responsible for aortic aneurysm and may ultimately lead to aortic dissection. Although the final consequences of LV-GCA may differ from C-GCA, the cellular infiltrates are largely similar. Infiltrating leukocytes in the inflamed aorta largely dominate the adventitia and the media layer of the aorta. Infiltration of both activated CD4+ and CD8+ T cells has been reported in GCA affected aorta (51). While the infiltration of T cells in adventitia and media of the aorta has been described, infiltration of CD20+ B cells mainly localizes in the adventitia where these cells are organized into TLOs (38). Macrophages expressing CD64, CD86, CD206, and FRβ are abundant in the adventitia and the media of GCA-affected aortas (41). CD206+/MMP-9+/YKL-40+ macrophages surround the necrotic areas in the media indicating a role in medial destruction (41). Notably, a reduction of α-SMA+ cells has been reported in the media of GCA-affected aortas due to medial necrosis which differs from the increase of adventitial and intimal α-SMA+ myofibroblasts in temporal arteries (52).

### PMR

In contrast to GCA, not much is currently known regarding the immunopathology of PMR. One of the first reports studying synovial tissue biopsies of PMR patients was published in 1964 in which hyperplasia of synovial lining cells, increased vascularity, and leukocyte infiltration mainly consisting of lymphocytes and macrophages were described (53). In the 1990s, immunohistological investigation on glenohumeral synovial tissue of patients with PMR revealed that CD68+ macrophages comprise the majority of the infiltrating cells followed by T cells and a small percentage of neutrophils (54). The T cell infiltrates were mainly comprised of CD45RO+ memory CD4+ T cells although small numbers of CD8+ T cells were also detected. B cells were not detected in the synovial tissue. In another report, the same group showed elevated VEGF expression by both CD3+ T cells and CD68+ macrophages in synovial biopsy tissues which correlated with vessel density indicating that these cells are involved in vasa vasorum formation and subsequently enhanced leukocyte infiltration in the synovium of PMR patients (55). More recently, enrichment of both CD4+ and CD8+ T cells in the synovial fluid of PMR patients have been reported (56). These T cells show a high IFNγ producing capacity pointing toward Th1 and Tc1 subsets. To date, no further cellular profiling has been done in the synovial tissues of PMR patients. Taking clues from other inflammatory diseases of the joint such as rheumatoid arthritis (RA) and osteoarthritis (OA), infiltration of CD206+, FRβ+, CD163+, MMP-9+ and iNOS+ macrophages has been reported (57, 58). Whether or not these cells are also involved in the immunopathology of PMR remains to be elucidated. Therefore, it is warranted for future studies to focus on deeper phenotyping of the cellular infiltrates in PMR synovial biopsies to better understand their roles in the immunopathology of PMR and subsequently target these cells for imaging and therapeutic purposes.

## Potential Novel PET Tracers for Diagnosis and Monitoring of GCA and PMR

The knowledge regarding the cellular heterogeneity in the pathogenesis of GCA and PMR may allow us to target these specific cells for imaging purposes. For a long time, [^18^F]FDG-PET has dominated the imaging landscape in oncology and inflammatory diseases alike. However, more recent efforts have shifted the trend toward targeting a specific cellular population. Targeting specific cell populations may provide the following advantages compared to [^18^F]FDG-PET:

(1) Lower background activity, thereby increasing target-to-background ratio and the imaging accuracy.

(2) Ability to evaluate specific cell populations as prognostic markers for disease progression and treatment response, thereby aiding the design of personalized treatment regimens, especially with emerging novel immunotherapies.

(3) Better insight into treatment effects. Novel immunotherapies often specifically target cellular pathways which may alter specific cell populations at the site of inflammation.

(4) Cellular infiltrates are unlikely to disappear immediately upon initiation of treatments in patients with immune-mediated inflammatory diseases including GCA and PMR (54, 59, 60), whereas especially glucocorticoid treatment may promptly impact the glucose metabolism by infiltrating immune cells and liver. Thus, the time interval of diagnostic scanning for cell-specific PET tracers might potentially be longer.

**Figure 1:**
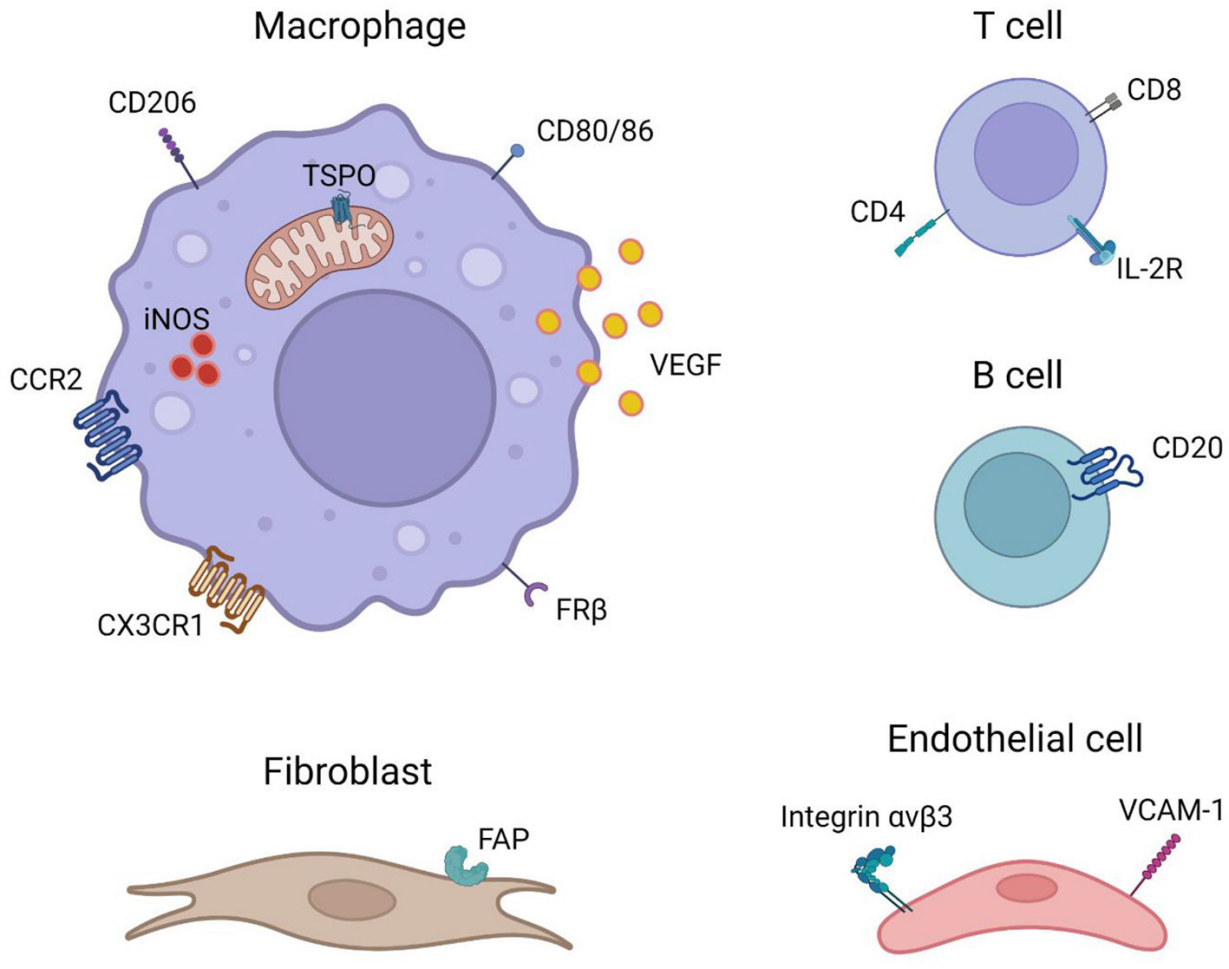
Imaging targets that are potentially useful for the imaging of GCA and PMR (created with BioRender.com).

As the efforts in developing PET radiotracers targeting specific cell populations are increasing, these radiotracers may also prove to be useful for the imaging of GCA and PMR which is summarized below (Figure 1).

### T Cell-Targeted Radiotracers

T cells are one of the most abundant infiltrating cell types in the inflamed GCA vessels (61). In the synovium of PMR patients, although not the most abundant cells, infiltration of T cells has been documented as well (54). Several radiotracers targeting T cells have been developed and are currently undergoing clinical trials for imaging other diseases, primarily oncology (Table 1). These radiotracers may also prove to be useful for the imaging of GCA and PMR patients.

**Table 1:** T cell targeted PET radiotracers.

**Target**	**Target marker/**	**Radiotracer**	**Class**	**Clinical/**	**Active clinical**	**References**
**cell**	**pathway**			**preclinical**	**trials**	
Activated T cells	IL-2R	[^18^F]FB-IL-2	Cytokine	Clinical	NCT04163094 NCT02478099	(62–64)
	IL-2R	[^18^F]AIF-RESCA-IL2	Cytokine	Preclinical	–	(65)
	IL-2R	[^68^Ga]Ga-NODAGA-IL2	Cytokine	Preclinical	–	(65)
CD4+ T cells	CD4 molecule	[^64^Cu]CD4-Nb1	Nanobody	Preclinical	–	(66)
		[^64^Cu]NOTA-IAB41	Minibody	Preclinical	–	(67)
CD8+ T cells	CD8 molecule	[^89^Zr]-Df-IAB22M2C	Minibody	Clinical	NCT03802123 NCT04874818 NCT04826393 NCT05013099 NCT04606316 NCT04180215 NCT03533283	(68–71)

IL-2 is a pleiotropic cytokine highly secreted by activated T cells which promotes T cell survival, expansion and differentiation into effector cells (72). The IL-2 receptor consists of three subunits including IL-2Rα (CD25), IL-2Rβ (CD122), and IL-2Rγ (CD132). IL-2 signals through the intermediary IL-2 receptor comprising the IL-2Rβ and IL-2Rγ chain. Upon activation, T cells gain elevated expression of CD25, completing the high-affinity receptor with the three subunits (72, 73). As a crucial cytokine in T cell functions, IL-2 is rapidly consumed by activated T cells making it an attractive cytokine for targeted imaging of activated T cells. The IL-2 targeted SPECT radiotracers, [^99m^Tc]IL-2 and [^99m^Tc]HYNIC-IL-2, have already been applied successfully for the visualization of vulnerable atherosclerotic plaques, transplant rejection and autoimmune thyroid disease (74–76). Furthermore, visualization of Takayasu arteritis has been reported in a case study utilizing [^99m^Tc]IL-2 scintigraphy (77), pointing toward the possible utility of IL-2 based lymphocyte targeted imaging in the detection of GCA. More recently, several PET radiotracers based on radionuclide tagged IL-2 have been reported. The first-generation IL-2 tracer, [^18^F]FB-IL-2, was reported to show high-affinity binding to activated human peripheral blood mononuclear cells (hPBMCs). The reports showed a high correlation of [^18^F]FB-IL-2 uptake with the number of CD25+ cells *in vitro* and in matrigel implants with activated hPBMCs (62–64). In a recent study, [^18^F]FB-IL-2 imaging successfully detected tumor lesions in metastatic melanoma patients (63). Biodistribution data showed high uptake in secretion organs (liver and kidneys), lymphoid organs (spleen and bone marrow) and the blood pool (myocardial and aortic) but low uptake in other non-target organs including the brain. The high blood pool radioactivity, however, may mask the detection of arterial inflammation in GCA. Recently, second-generation IL-2 based tracers, [^18^F]AIF-RESCA-IL2 and [^68^Ga]Ga-NODAGA-IL2 have been developed (65). Although yet to be tested in humans, both radiotracers showed high specific uptake in lymphoid tissue and hPBMC xenografts in a mouse model. In addition, both second-generation radiotracers showed no brain uptake and lower blood pool radioactivity compared to [^18^F]FB-IL-2 which may be advantageous for the detection of aortic and arterial inflammation in GCA. Furthermore, as T cell infiltration in the synovium of PMR patients has been documented, these radiotracers may also prove to be useful for PMR imaging.

Dominant CD4+ T cell infiltration over CD8+ T cells at the site of inflammation has been reported for both GCA and PMR (51, 54), making CD4+ T cells an attractive target for imaging of these diseases. Two ImmunoPET tracers targeting human CD4 T cells have been recently reported. Nanobody-based [^64^Cu]CD4-Nb1 showed specific uptake in organs with high numbers of CD4+ T cells including lymph nodes, thymus, spleen, and liver with rapid blood and lung clearance *via* renal elimination in a human CD4 knock-in mouse model (66). Similarly, minibody based [^64^Cu]NOTA-IAB41 showed specific uptake in CD4+ T cells infiltrated lungs, spleen, liver and kidney in hPBMC injected humanized mice (67). Interestingly, the report also showed successful visualization of CD4+ T cell infiltration in a humanized brain tumor mouse model compared to no brain uptake in the non-disease control group. Both radiotracers may potentially be useful in imaging GCA and PMR patients.

Lower numbers of infiltrating CD8+ T cells compared to CD4+ T cells have been reported in the inflamed vessels of GCA patients. However, CD8 targeted imaging may still be valuable for this disease since the presence of large arterial CD8 T cell infiltrates is associated with disease severity (51). A minibody based CD8+ T cell-targeted radiotracer, [^89^Zr]Df-IAB22M2C, has been developed and is currently actively investigated in several clinical trials. Reports of CD8+ T cells imaging in patients with solid tumors have shown successful visualization of tumor-infiltrating CD8+ T cells and specific uptake in CD8+ rich lymphoid organs (70, 71). Moreover, low blood pool radioactivity and no brain uptake were detected in these patients making it suitable for imaging C-GCA and LV-GCA. The infiltration of CD8+ T cells in the synovium of PMR patients is scarce and therefore CD8+ T cell-targeted imaging may be less suitable for PMR.

Although PD-1 expressing T cell infiltration has been reported in GCA (30, 31), only two radiotracers targeting PD-1 based on PD-1 antagonist nivolumab and pembrolizumab are currently available (78). Since the development of both GCA and PMR as consequences of PD-1/PD-L1 inhibition therapy of cancer patients has been reported (79, 80), the use of these radiotracers for the imaging of GCA and PMR may potentially worsen the disease and is therefore not feasible. However, with the rise of PD-1 agonists (81–84), future applications of these PD-1 agonist-based radiotracers may prove to be useful as a theranostic approach in these diseases.

### Macrophage Targeted Radiotracers

Macrophages play vital roles and are one of the most abundant cell types infiltrating the inflamed vessels and synovial tissue of GCA and PMR patients making them attractive targets for cell-specific imaging (5, 54). As macrophages are incredibly plastic cells, not all tracers are suitable for the imaging of macrophages in GCA and PMR patients. As our knowledge regarding macrophage heterogeneity in the pathogenesis of GCA has improved considerably, we will focus on a selected number of radiotracers targeting macrophage phenotypes that have been proven to be involved in the vasculopathy of GCA (Table 2).

**Table 2:** Macrophage targeted PET radiotracer.

**Target marker/**	**Radiotracer**	**Class**	**Clinical/**	**Active clinical**	**References**
**pathway**			**preclinical**	**trials**	
TSPO	[^11^C]PK11195	TSPO antagonist	Clinical	NCT03368677	(85–94)
				NCT04239820	
				NCT05111678	
	[^11^C]-(R)-PK11195	TSPO antagonist	Clinical	–	(95–114)
	[^11^C]DAA1106	TSPO agonist	Clinical	–	(115–121)
	[^18^F]DAA1106	TSPO agonist	Preclinical	–	(122–124)
	[^18^F]FEDAA1106	TSPO agonist	Clinical	–	(125–130)
	[^18^F]FMDAA1106	TSPO agonist	Preclinical	–	(125)
	[^11^C]PBR28	TSPO agonist	Clinical	NCT04274998 NCT05205291 NCT04230174 NCT03705715 NCT04490096 NCT02649985 NCT04236986 NCT04398719 NCT04811404 NCT04066244 NCT04233593 NCT03106740 NCT05066308 NCT04486118 NCT05183087	(131–145)
	[^18^F]PBR06	TSPO agonist	Clinical	NCT04510220 NCT03983252 NCT04144257 NCT02649985	(146–150)
	[^11^C]PBR06	TSPO agonist	Preclinical	–	(147)
	[^11^C]PBR01	TSPO agonist	Preclinical	–	(151, 152)
	[^18^F]FEPPA	TSPO agonist	Clinical	NCT02945774 NCT04814355 NCT04307667 NCT04854785	(153–163)
	[^18^F]PBR111	TSPO antagonist	Clinical	–	(164–169)
	[^18^F]PBR102	TSPO antagonist	Preclinical	–	(166, 169, 170)
	[^11^C]CB184	TSPO antagonist	Clinical	–	(171–174)
	[^11^C]CLINME	TSPO ligand	Preclinical	–	(167, 175, 176)
	[^18^F]CB251	TSPO ligand	Preclinical	–	(177–179)
	[^11^C]AC-5216	TSPO agonist	Preclinical	–	(180)
	[^11^C]DAC	TSPO agonist	Preclinical	–	(181, 182)
	[^11^C]DPA-713	TSPO agonist	Clinical	–	(183–193)
	[^18^F]DPA-714	TSPO agonist	Clinical	NCT03759522 NCT03754348 NCT03457493 NCT04362644 NCT03999164 NCT04364672 NCT04542603 NCT03230526 NCT03968445 NCT05238961 NCT04520802 NCT05147532 NCT03691077 NCT04171882 NCT03482115 NCT03314155	(193–195)
				NCT05128903 NCT03435861 NCT05233605 NCT04785157	
	[^18^F]DPA-C5yne	TSPO agonist	Preclinical	–	(196)
	[^18^F]FDPA	TSPO agonist	Preclinical	–	(197–200)
	[^18^F]VUIIS1018A	TSPO agonist	Preclinical	–	(201, 202)
	[^18^F]FEDAC	TSPO ligand	Preclinical	–	(203–205)
	[^11^C]SSR180575	TSPO agonist	Preclinical	–	(206)
	[^18^F]SSR180575	TSPO agonist	Preclinical	–	(207)
	[^18^F]FEMPA	TSPO ligand	Clinical	NCT05039268	(208–210)
	[^18^F]FETEM	TSPO ligand	Preclinical	–	(211)
	[^18^F]FEBMP	TSPO ligand	Preclinical	–	(212, 213)
	[^18^F]GE180	TSPO ligand	Clinical	NCT04412187 NCT03702816	(214–223)
	(R)-[^18^F]NEBIFQUINIDE	TSPO ligand	Preclinical	-	(224)
	[^11^C]ER176	TSPO ligand	Clinical	NCT04762719 NCT04840979 NCT03705715 NCT04576793 NCT03958630 NCT03912428 NCT04510168 NCT04786548	(225–228)
	[^18^F]ER176 analogs	TSPO ligand	Preclinical	–	(229, 230)
	[^18^F]-(S)-GE387	TSPO ligand	Preclinical	–	(223, 231)
	[^18^F]LW223	TSPO ligand	Preclinical	–	(232, 233)
	[^18^F]BS224	TSPO ligand	Preclinical	–	(234)
CD206	[^64^Cu]MAN-LIPs	Mannose coated liposome	Preclinical	–	(235)
	[^68^Ga]NOTA-MSA	Mannosylated protein	Clinical	–	(236–239)
	[^18^F]FDM	Mannose derivative	Clinical	–	(240, 241)
	[^68^Ga]IRDye800-tilmanocept	Mannose derivative	Preclinical	–	(242)
	[^18^F]FB-anti-MMR 3.49 sdAb	Nanobody	Preclinical	–	(243)
	[^68^Ga]MMR	Nanobody	Preclinical	–	(244)
	[^68^Ga]Ga-NOTA-anti-MMR-sdAb	Nanobody	Preclinical	–	(245)
	[^68^Ga]Ga-NOTA-MMR Nb	Nanobody	Preclinical	–	(246)
	[^68^Ga]Ga-NOTA-anti-MMR-VHH2	Nanobody	Clinical	NCT04168528 NCT04758650	–
FRβ	[^18^F]α/γ-FBA-Folate	Folate derivative	Preclinical	–	(247)
	[^18^F]click-folate	Folate derivative	Preclinical	–	(248)
	[^68^Ga]DOTA-folate	Folate derivative	Preclinical	–	(249)
	2'-[^18^F]fluorofolic acid	Folate derivative	Preclinical	–	(250)
	[^18^F]fluorobenzene-folate	Folate derivative	Preclinical	–	(251)
	[^18^F]pyridinecarbohydrazide-folate	Folate derivative	Preclinical	–	(251)
	[^18^F]fluorodeoxy-glucose-folate	Folate derivative	Preclinical	–	(252–254)
	[^68^Ga]NODAGA-folate	Folate derivative	Preclinical	–	(255)
	[^68^Ga]DO3A-EA-Pte	Pteroic acid derivative	Preclinical	–	(256)
	3′-aza-2′[^18^F]fluorofolicacid/[^18^F]AzaFol	Folate derivative	Clinical	–	(257–259)
	[^18^F]fluoro-PEG-folate	Folate derivative	Clinical	NCT05215496	(260–263)
	4-[^18^F]-fluorophenylfolate	Folate derivative	Preclinical	–	(264)
	[^68^Ga]NOTA-folate	Folate derivative	Preclinical	–	(265, 266)
	α/β-click[^18^F]FDG-folate	Folate derivative	Preclinical	–	(267)
	α/β-click[^18^F]FE-folate	Folate derivative	Preclinical	–	(267)
	α/β-click[^18^F]FB-folate	Folate derivative	Preclinical	–	(267)
	Folate-NOTA-Al[^18^F]	Folate derivative	Preclinical	–	(268)
	[^64^Cu]rf42	Folate derivative	Preclinical	–	(269)
	[^68^Ga]rf42	Folate derivative	Preclinical	–	(269)
	[^68^Ga]DOTA-Lys-Pteroyl	Pteroyl-Lys derivatives	Preclinical	–	(270)
	[^68^Ga]DOTA-DAV-Lys-Pteroyl	Pteroyl-Lys derivatives	Preclinical	–	(270)
	[^68^Ga]NOTA-folic acid	Folate derivative	Preclinical	–	(271)
	[^89^Zr]FA-DFO-liposome	Folate coated liposome	Preclinical	–	(272)
	[^68^Ga]HCEF	Folate derivative	Preclinical	–	(273)
	Folate-PEG12-NOTA-Al[^18^F]	Folate derivative	Preclinical	–	(274)
	[^64^Cu]DOTA-FA-FI-G5·NHAc dendrimers	Folate tagged dendrimers	Preclinical	–	(275)
	[^18^F]Ala-folate	Folate derivative	Preclinical	–	(276)
	[^18^F]DBCO-folate	Folate derivative	Preclinical	–	(276)
	[^18^F]FOL	Folate derivative	Preclinical	–	(277, 278)
	[^68^Ga]NODAGA-FA	Folate modified polymer	Preclinical	–	(279)
	[^55^Co]Co-cm10	Folate derivative	Preclinical	–	(280)
	[^55^Co]Co-rf42	Folate derivative	Preclinical	–	(280)
	[^68^Ga]Ga-FA-I	Folate derivative	Preclinical	–	(281)
	[^68^Ga]Ga-FA-II	Folate derivative	Preclinical	–	(281)
	[^89^Zr]SFT-1	Folate coated nanoparticle	Preclinical	–	(282)
	[^68^Ga]FOL	Folate derivative	Preclinical	–	(283)
	[^89^Zr]FA-SMWs	Folate coated micro-silica	Preclinical	–	(284)
CD80/86	[^64^Cu]NODAGA-abatacept	Biologic	Preclinical	–	(285)
iNOS	S-[^11^C]methylisothiourea	iNOS inhibitor	Preclinical	–	(286)
	S-(2-[^18^F]fluoroethyl)-isothiourea	iNOS inhibitor	Preclinical	–	(286)
	[^18^F]FFDI	iNOS inhibitor	Preclinical	–	(287)
	[^18^F]6-(2-fluoropropyl)-4-methyl-pyridin-2-amine	iNOS inhibitor	Preclinical	–	(288)
	[^18^F]NOS	iNOS inhibitor	Clinical	NCT04062526	(289, 290)
	[^18^F]FBAT	iNOS inhibitor	Preclinical	–	(291)
CCR2	[^64^Cu]DOTA-ECL1i	Peptide	Clinical	NCT04217057 NCT03492762 NCT05107596 NCT04592991 NCT04537403 NCT03851237 NCT04586452	(292–300)
	[^64^Cu]AuNCs-ECL1i	Peptide	Preclinical	–	(293)
	[^64^Cu]Cu@CuO_x_	Nanoparticle	Preclinical	–	(301)
	[^18^F]6b	Chemical compound	Preclinical	–	(302)
CX3CR1	[^11^C]methyl(2-amino-5(benzylthio)thiazolo[4,5-d]pyrimidin-7-yl)-d-leucinate	Chemical compound	Preclinical	–	(303)
VEGF	[^124^I]HuMV833	Antibody	Clinical	–	(304)
	[^124^I]iodinated-VG76e	Antibody	Preclinical	–	(305)
	[^89^Zr]bevacizumab	Antibody	Clinical	–	(306–317)
	[^64^Cu]DOTA-bevacizumab	Antibody	Preclinical	–	(318)
	[^89^Zr]ranibizumab	Antibody	Preclinical	–	(319)
	[^64^Cu] NOTA-Bev-800CW	Antibody	Preclinical	–	(320)
	[^124^I]aflibercept	VEGF antagonist	Preclinical	–	(321)
	[^64^Cu]NOTA-bevacizumab	Antibody	Preclinical	–	(322)
	[^64^Cu]L19K-FDNB	Peptide	Preclinical	–	(323)

Macrophage-targeted imaging of translocator protein (TSPO; also known as PBR or peripheral benzodiazepine receptor) was first developed for the targeted imaging of microglia in neuroinflammation. It was later found that TSPO targeted imaging could be utilized for the imaging of non-neuronal inflammatory diseases such as rheumatoid arthritis (RA), atherosclerosis, Takayasu arteritis (TAK), systemic lupus erythematosus (SLE), and GCA (88, 94, 98–100, 103, 106, 185, 190, 193). In 2010, Pugliese et al. successfully showed the utility of [^11^C]PK11195, a first-generation TSPO targeted radiotracer, in the detection of large-vessel vasculitis including GCA (94). Shortly after, another study led by Lamare et al. utilizing the radiotracer [^11^C]-(R)-PK11195 showed a similar result in detecting vascular inflammation in patients suffering from large-vessel vasculitis (106). Additionally, studies have shown high uptake of a second-generation TSPO tracer, [^11^C]DPA-713, in inflamed joints of RA patients pointing to the potential in imaging PMR (185, 193). Although the utility has been shown in imaging GCA, older generation TSPO targeted radiotracers possess several disadvantages. The major drawback of these radiotracers is the significantly lower binding capability to TSPO in patients with a common TSPO gene polymorphism (*rs6971*), which resulted in variability in the imaging signal (324, 325). Furthermore, these older generation TSPO targeted tracers are not very specific and show high background radioactivity, which may hamper the imaging capability (326). The third generation of TSPO targeted radiotracers, such as [^18^F]FEBMP, [^18^F]ER176 and [^18^F]-(S)-GE387, are designed to overcome these drawbacks. Notably, a recent study using another second-generation tracer, [^11^C]PBR28, documented no vascular uptake in patients suffering from large-vessel vasculitis (Takayasu arteritis and GCA) (327). It is unclear whether this discrepancy was caused by the unspecific binding of the first-generation TSPO tracers in the inflamed tissues. This discrepancy warrants a more detailed investigation into the feasibility of new generation TSPO targeted tracers in imaging GCA.

The mannose receptor (CD206) is a C-type lectin receptor highly expressed by certain populations of macrophages. Several CD206 targeted radiotracers have been developed and some have been used in clinical applications (Table 2). Employing the receptor-ligand binding approach, tracers based on mannose derivatives, mannose coated liposome, and mannosylated protein have shown reliable CD206 targeting. [^18^F]FDM based on fluorodeoxy-mannose have been shown to have lower non-specific brain uptake compared to [^18^F]FDG in patients with brain infarction (241). Furthermore, this radiotracer has been successfully used in preclinical imaging of atherosclerotic lesions in a rabbit model supporting its potential in vascular imaging (328). More recently, anti-CD206 nanobody-based radiotracers have been developed which are more specific compared to the mannose derivatives-based tracers. In animal models of atherosclerosis and cancer, these radiotracers have shown rapid blood clearance and low radioactivity in non-target organs including the brain (243, 245, 246). The abundance of CD206+ macrophages responsible for expressing high levels of YKL-40 and MMP-9, a proangiogenic and tissue destructive collagenase, respectively, has been documented in affected vessels of GCA patients (41, 42). These macrophages are likely skewed by GM-CSF in the microenvironment and are considered to be responsible for media destruction in GCA. Given their prominent roles, targeted imaging of CD206+ macrophages may be beneficial for the diagnosis and prognosis of GCA.

The interest in folate-based imaging started over two decades ago when folate receptor-expressing tumors were discovered (329). This led to the rapid development of numerous folate receptor-targeted radiopharmaceuticals. Of note, some of these radiotracers are tagged with highly radioactive nuclides or toxic particles intended as radiotherapeutics for cancers (330–333). More recently, it has been demonstrated that some macrophages involved in autoimmune and inflammatory diseases, including GCA, express high levels of folate receptor beta (FRβ) as well. Interestingly, the degree of FRβ+ macrophage infiltration in the intima of the GCA affected vessels has been linked to intimal hyperplasia (41). Patients with intimal hyperplasia in their biopsy may be more likely to develop ischemic complications than those without (334). Imaging FRβ may potentially reveal the degree of vessel wall occlusion in C-GCA and may signal the need for the rapid induction of high-dose GC therapy to lower the risk of vision loss in these patients. Among the plethora of FR-targeted radiotracers, [^18^F]fluoro-PEG-folate and [^18^F]AzaFol have been used in clinical applications (259, 263). [^18^F]fluoro-PEG-folate was assessed in patients with RA and showed specific uptake in inflamed joints with low brain uptake. The infiltration of FRβ+ macrophages in the synovial tissue of RA patients has been well-documented. On the other hand, the utility of [^18^F]AzaFol was assessed in patients with FRα+ adenocarcinoma of the lungs. These studies demonstrate the utility of these novel folate receptor-targeted radiotracers in human subjects, but at the same time revealed that folate receptor imaging may not be very specific due to uptake by both FRα+ and FRβ+ cells. Nevertheless, folate receptor imaging may still prove to be useful in patients suffering from GCA especially in the detection of patients at risk of developing ischemic events due to severe intimal hyperplasia.

Immunotherapy targeting T cell activation by blocking CD80/86 on antigen-presenting cells (APCs) with a CTLA-4Ig fusion protein (abatacept) is currently evaluated in GCA (NCT04474847). An earlier phase II randomized control trial of abatacept for the treatment of GCA showed promising results with significant proportions of patients achieving relapse-free survival rate at 12 months compared to placebo (48% abatacept vs. 31% placebo; *p* = 0.049) (335). Separately, a case report has shown the potential application of abatacept for the treatment of PMR (336). Together, these encouraging results may point toward the potential application of radionuclide tagged [^64^Cu]NODAGA-abatacept in GCA and PMR.

Inducible nitric oxide synthase (iNOS) is a reactive oxygen and nitrogen metabolite-metabolizing enzyme typically expressed by activated proinflammatory macrophages. The utility of [^18^F]NOS, iNOS targeted radiotracer based on iNOS inhibitor has been reported in allograft rejection patients and patients with acute lung inflammation (289, 290). The studies also showed low brain radioactivity suitable for the imaging of C-GCA. Intimal infiltrating iNOS+ macrophages have been previously reported in GCA whereas in the adventitia of these vessels iNOS+ macrophages were absent (48). Therefore, iNOS imaging may be valuable as a tool to detect intimal macrophage infiltration and potentially intimal hyperplasia.

The chemokine receptors CCR2 and CX3CR1 are responsible for the trafficking of monocytes into the GCA affected vessel wall where these cells will then mature into macrophages (33). The radiotracer [^64^Cu]DOTA-ECL1i specifically targeting CCR2 may be useful for imaging infiltrating monocytes/macrophages in GCA affected vessels. The utility of this radiotracer has recently been investigated in patients with pulmonary fibrosis (298). The study showed specific uptake in diseased lungs with little uptake in healthy controls. Moreover, low non-specific brain uptake and low blood radioactivity may be beneficial for imaging both LV-GCA and C-GCA. On the other hand, the radiotracer [^11^C]methyl(2-amino-5(benzylthio)thiazolo[4,5-d]pyrimidin-7-yl)-d-leucinate designed to target CX3CR1, failed to show specific binding to CX3CR1 and therefore is not suitable for imaging GCA at the current state (303). As CX3CR1+ monocyte infiltration was reported to be higher than CCR2+ monocytes, future radiotracers targeting CX3CR1 may be beneficial for the imaging of GCA.

The abundance of vascular endothelial growth factor (VEGF), a potent pro-angiogenic growth factor, has been reported in the synovium of PMR patients (55). Macrophages have been implicated as the major source of VEGF as these cells are the major cellular infiltrates in the inflamed synovium (55). The antibody-based radiotracer [^89^Zr]bevacizumab targeting VEGF has been successfully used to visualize VEGF expression in multiple oncological conditions (308, 314–317). Additionally, the utility of [^89^Zr]bevacizumab in detecting VEGF expression in atherosclerotic plaques has been shown in *ex vivo* imaging studies of human carotid endarterectomy (CEA) specimens (309). Since the increased expression of VEGF has been reported in PMR, [^89^Zr]bevacizumab imaging may be useful for imaging PMR patients. Of note, although inflammatory macrophages are major producers of VEGF in PMR, infiltrating T cells are also capable of producing VEGF (55). Hence, VEGF-targeted imaging may not be specific for macrophages. In GCA, heightened VEGF expression has been documented especially in the adventitia of GCA-affected vessels (337). However, whether macrophages or T cells are the main producers of VEGF in GCA lesions remains to be further explored. Nevertheless, VEGF imaging may potentially also be useful for imaging GCA.

Most of the macrophage-targeted radiotracers discussed above may be suitable for imaging GCA. However, whether similar macrophage phenotypes are involved in the pathogenesis of PMR remains to be proven since, to our knowledge, no study to date has explored macrophage heterogeneity in PMR.

### B Cell-Targeted Radiotracers

B cell infiltration and organization into TLOs have been well-documented in GCA (38, 338). However, B cell appear to be absent in the synovial tissue of PMR patients (54). Therefore, B cell-targeted imaging may only be suitable for GCA. Several B cells targeted radiotracers have been developed (Table 3).

**Table 3:** B cell targeted PET radiotracers.

**Target marker/**	**Radiotracer**	**Class**	**Clinical/**	**Active clinical**	**References**
**pathway**			**preclinical**	**trials**	
CD20	[^64^Cu]DOTA-minibody (based on rituximab)	Minibody	Preclinical	–	(339)
	[^124^I]anti-CD20-Cys-Db (based on rituximab)	Diabody	Preclinical	–	(340)
	[^124^I]rituximab	Antibody	Clinical	–	(341, 342)
	[^89^Zr]rituximab	Antibody	Clinical	–	(343–348)
	[^64^Cu]DOTA-rituximab	Antibody	Preclinical	–	(349, 350)
	[^64^Cu]FN3(CD20)	Protein	Preclinical	–	(351)
	[^18^F]FB-GAcDb (based on obinutuzumab)	Diabody	Preclinical	–	(352)
	[^89^Zr]GacDb (based on obinutuzumab)	Diabody	Preclinical	–	(353)
	[^89^Zr]GacMb (based on obinutuzumab)	Minibody	Preclinical	–	(353)

A case report has documented the resolution of vascular inflammation in a GCA patient with rituximab B cell depletion therapy (354). However, no further trials are currently ongoing for rituximab therapy in GCA. Rituximab-based radiotracer [^89^Zr]rituximab has been successfully used to image B cells in lymphoma and RA patients (347, 348). The radiotracer showed low background activity in the blood pool which may support its suitability for application in imaging GCA.

### Activated Fibroblast Targeted Radiotracers

Remodeling of the arterial wall secondary to inflammation may cause vessel occlusion and hence, be responsible for the ischemic events in GCA. Fibroblast activation, migration, and proliferation in the intima have been reported as one of the causes of intimal hyperplasia (50). Currently, the targeted imaging of fibroblast activation protein alpha (FAP), a serine protease expressed mainly by activated fibroblast, is gaining tremendous interest in cancer and inflammatory diseases (355). The interest in FAP targeted imaging started with the development of radiolabeled FAP inhibitor [^125^I]MIP-1232 for single-photon emission computed tomography (SPECT) imaging of atherosclerosis (356). However, *ex vivo*, the radiotracer showed uptake in normal arteries as well hampering its utility for atherosclerotic imaging. Since then, more specific FAP inhibitors (FAPIs) have been rapidly developed and radiolabeled as PET radiotracers (Table 4).

**Table 4:** Fibroblast activation protein alpha (FAP) targeted PET radiotracers.

**Radiotracer**	**Class**	**Clinical/preclinical**	**Active clinical trials**	**References**
[^68^Ga]FAPI-02	FAP inhibitor	Preclinical	–	(357)
[^68^Ga]DOTA.SA.FAPI	FAP inhibitor	Clinical	–	(358, 359)
[^68^Ga]FAPI-04/[^68^Ga]DOTA-FAPI-04	FAP inhibitor	Clinical	NCT05003427 NCT04504110 NCT04533828 NCT04441606 NCT04499365 NCT04831034 NCT04416165 NCT05121779 NCT05140746	(357)
[^68^Ga]NOTA-FAPI-04	FAP inhibitor	Clinical	NCT04499365 NCT04367948 NCT04750772 NCT05004961	(360)
[^68^Ga]FAPI-21	FAP inhibitor	Clinical	–	(361)
[^68^Ga]FAPI-46	FAP inhibitor	Clinical	NCT05160051 NCT04941872 NCT04457258 NCT04457232 NCT04459273 NCT04147494 NCT05172310	(361)
[^68^Ga]TEFAPI-06	FAP inhibitor	Preclinical	–	(362)
[^68^Ga]TEFAPI-07	FAP inhibitor	Preclinical	–	(362)
[^68^Ga]FAPI-C12	FAP inhibitor	Preclinical	–	(363)
[^68^Ga]FAPI-C16	FAP inhibitor	Preclinical	–	(363)
[^68^Ga]FAPtp	FAP inhibitor	Preclinical	–	(364)
[^68^Ga]Aib-FAPtp-01	FAP inhibitor	Preclinical	–	(364)
[^68^Ga]DOTA-2P(FAPI)_2_	FAP inhibitor	Clinical	NCT04941872	(365)
[^18^F]FGlc-FAPI	FAP inhibitor	Preclinical	–	(366)
Al[^18^F]NOTA-FAPI	FAP inhibitor	Clinical		(367)
[^18^F]NOTA-FAPI-04	FAP inhibitor	Clinical	NCT04367948 NCT04750772 NCT05004961	–

From these FAPI based radiotracers, [^68^Ga]FAPI-04 has been rapidly implemented in clinical trials and has shown superiority compared to the long-time gold standard [^18^F]FDG for imaging cancer and inflammation as recently summarized by Li et al. (355). These reports showed high and specific uptake in tumors as well as at sites of fibrosis and inflammation while displaying negligible blood pool and brain radioactivity supporting its potential application for imaging both C-GCA and LV-GCA. Although FAP expression has not yet been investigated in the context of GCA and PMR, a case report has shown successful visualization of aortic and arterial inflammation in a patient suffering from GCA using [^68^Ga]FAPI-04 (368). Interestingly [^68^Ga]FAPI-04 imaging showed negligible radioactivity in non-target organs including the brain, background tissue, and in the blood pool, which may allow accurate detection of C-GCA. This case report demonstrates the potential of FAP targeted imaging for visualization of vascular inflammation in GCA warranting further investigation especially in comparison with the current gold standard [^18^F]FDG-PET. No reports have shown the utility of FAP targeted imaging in PMR patients to date. Yet, successful FAP targeted imaging has been demonstrated in RA patients which may point to its potential to image inflammation in PMR patients as well (369).

### Endothelial Cell-Targeted Radiotracers

Neoangiogenesis is one of the crucial pathogenic features of GCA and PMR. Increased vascularity in the vessel wall and synovium of GCA and PMR patients further enables the invasion of leukocytes thereby fueling the inflammatory process (32, 54, 55). In uninflamed vessels, the luminal endothelium does not express the inducible adhesion molecules VCAM-1. In inflamed GCA-affected vessels, the intense expression of VCAM-1 has been reported on neovessel endothelial cells making this adhesion molecule suitable for targeted imaging (32). Several radiotracers targeting VCAM-1 have been developed (Table 5) which may be useful for imaging GCA.

**Table 5:** Adhesion molecule VCAM-1 targeted PET radiotracers.

**Target marker/**	**Radiotracer**	**Class**	**Clinical/**	**Active clinical**	**References**
**pathway**			**preclinical**	**trials**	
VCAM-1	[^18^F]4V	Peptide	Preclinical	–	(370)
	[^18^F]-FB-anti-VCAM-1 Nb	Nanobody	Preclinical	–	(371)
	[^68^Ga]NOTA-VCAM-1_scFv_	Antibody fragment	Preclinical	–	(372)
	[^68^Ga]MacroP	Peptide	Preclinical	–	(373)
	NAMP–avidin–[^68^Ga]–BisDOTA	Peptide	Preclinical	–	(373)

An alternative approach to image angiogenesis is to target the integrin αvβ3 and specific radiotracers for this integrin have already been applied in clinical practice for imaging tumor metastasis (374–376). However, whether this approach is suitable for imaging GCA is uncertain since the αvβ3 integrin is constitutively expressed on the luminal endothelium (377). Nevertheless, studies have demonstrated the enhanced uptake of αvβ3 integrin-targeted tracers in atherosclerotic plaques corresponding to neo-vessel formation indicating its potential utility in GCA (374, 378). Furthermore, imaging integrin αvβ3 may be of interest for PMR as increased vascularization has been reported in the synovial tissues from PMR patients. Of note, integrin αvβ3 imaging is not specific for angiogenesis as infiltrating leukocytes can also express this adhesion molecule (379).

## Future Perspectives: Toward Disease Stratification and Better Treatment Monitoring

The molecular PET imaging technique targeting specific markers has made valuable contributions to clinical practice ranging from diagnosis, staging, and prognosis to treatment monitoring. There are clear examples in other fields of medicine, mainly from the field of oncology, supporting the use of targeted imaging in patient stratification for targeted therapy. For example, anti-human epidermal growth factor receptor 2 (HER2) targeted therapy is only effective in HER2+ breast cancer accounting for only up to 30% of newly diagnosed breast cancer patient which can be visualized by HER2 targeted PET imaging (380–383). In another study, HER2+ PET imaging using [^89^Zr]trastuzumab in combination with [^18^F]FDG resulted in a negative and positive predictive value of 100% for discriminating between patients with a time to treatment failure of 2.8 and 15 months (384). A recent preclinical study in a cancer mouse model, *in vivo* imaging of different receptor tyrosine kinases (RTKs) demonstrated a decrease in receptor expression levels after their respective targeted therapy (385). Beyond oncology, [^18^F]FDOPA PET imaging of striatal dopaminergic system has been shown to effectively stratify responders and non-responders of antipsychotic treatment in schizophrenic patients (386). Collectively, these studies support the utility of targeted PET imaging in aiding patient stratification for specific treatment strategies, prognosis and precision monitoring of treatment effect in inflammatory diseases including GCA and PMR.

In imaging GCA and PMR, radiotracers targeting specific cell populations may potentially be superior compared to the current gold standard [^18^F]FDG. The majority of the novel PET radiotracers listed above have shown low non-target organ uptake, especially in the brain, which could increase the TBR and may translate into improved detection of cranial artery inflammation in patients suffering from C-GCA. Additionally, several novel radiotracers show low blood pool radioactivity assuring optimal TBR and visualization of aortic and arterial inflammation in LV-GCA.

Since persistent T cell and macrophage infiltration has been reported in TAB of patients undergoing glucocorticoid treatment (59, 60), imaging T cell and macrophage subsets could, in theory, be superior to [^18^F]FDG in the diagnostic imaging of GCA. Similarly, these radiotracers could also be useful for the diagnostic imaging of PMR as persistent T cell and macrophage infiltration has also been shown in PMR patients undergoing GC treatment (54).

Imaging specific leukocyte populations may have prognostic value and may help in designing personalized treatment regimens for GCA and PMR (387). The utility of immune cell targeted imaging has indeed been reported in the context of oncology and in autoimmune inflammatory diseases. An example of this was reported in a study conducted using [^99m^TC]IL-2 scintigraphy in melanoma patients. The study showed successful visualization of tumor infiltrating lymphocytes which enables the selection of patient whom may benefit from IL-2 immunotherapy (388). Another example was reported in patients with rheumatoid arthritis (RA) using B cell targeted [^89^Zr]rituximab (348). The study showed that patients who responded to B cells depletion therapy had higher baseline imaging signal. In the context of GCA, the higher intensity of CD8+ T cell infiltration in the vessel wall of GCA patients has been proposed as a risk factor for visual impairment and a longer GC treatment dependency. This suggests that CD8+ T cell imaging in GCA may confer prognostic value (35). In the B cell compartment, CD20-based imaging could prove to be useful as a theranostic approach to identify GCA patients that may benefit from rituximab treatment followed by a therapeutic dose of rituximab after imaging confirmation (354). In another example, we have previously reported the prognostic value of serum levels of YKL-40 in patients suffering from GCA (389). Higher levels of serum YKL-40 at baseline predicted a longer duration of GC treatment. In the GCA-affected vessel wall, YKL-40 is highly expressed by GM-CSF skewed CD206+ macrophages (42). Therefore, imaging the extent of CD206+ infiltration in the vessels may also predict the GC dependency of these patients. Recently, a phase II clinical trial with a GM-CSF receptor blocker (mavrlimumab) demonstrated GM-CSF receptor blockade to be efficacious in the treatment of GCA (390). Furthermore, *ex vivo* treatment of GCA-affected vessels with mavrilimumab documented a reduction of CD206 expression (40). Based on these studies, imaging CD206 in GCA patients may potentially identify patients that could benefit from mavrilimumab treatment. Along similar lines, the cytokine IL-6 has been reported to elevate the expression of CD163 on macrophages (5). Hence, the detection of CD163+ macrophages may reveal GCA patients that could benefit from the IL-6 receptor blocker, tocilizumab. Although no report has shown infiltration of CD163+ macrophages in PMR, IL-6 is a major cytokine involved in the pathogenesis of this disease denoting the possibility of CD163+ macrophage infiltration in the synovium of PMR patients. Unfortunately, the only CD163 targeted radiotracer currently reported was developed for preclinical imaging in rat models but does not cross-react with human CD163 (391). Future development of CD163 targeted tracers may be beneficial for the imaging of GCA patients and potentially PMR patients.

The novel radiotracers discussed in this review may also be used for monitoring treatment efficacy. Reduced numbers of T cells and macrophages at the site of inflammation have been reported (54, 59, 60). This may translate to a gradual decrease in imaging signal during treatment which could be useful for monitoring ongoing inflammation during GC treatment. Furthermore, the reduced expression of endothelial adhesion molecules VCAM-1 and E-selectin has been reported in the vessels of GCA patients undergoing GC treatment (32). In addition, targeted imaging of specific cell populations could also be used for monitoring the efficacy of novel immunotherapies. Reduced CD206 expression and neovascularization have been reported in *ex vivo* cultured temporal artery explants of GCA patients treated with mavrilimumab (40). Therefore, tracking the dynamics of these cellular markers by imaging may be useful for treatment effect monitoring in patients undergoing mavrilimumab treatment.

Although these novel radiotracers may be useful for imaging GCA and PMR, several considerations have to be taken into account before these tracers can be applied in clinical practice. Firstly, some of these novel tracers are tagged with radionuclides with high radiation doses such as ^89^Zr (392). Nuclides with high radioactivity are necessary for tracers based on large molecules with low tissue penetration rates such as antibodies. The long half-life of ^89^Zr (3.3 days) allows a longer period of time for effective tissue penetration and blood clearance to ensure that the signal can be imaged after a prolonged time frame after injection. This higher radiation dose is permissible in imaging oncology patients but is not recommended for patients with autoimmune and inflammatory diseases such as GCA and PMR. Therefore, it is important to develop radiotracers with better tissue penetration rates and tagged with radionuclides with lower radiation burdens such as ^18^F. The current emerging technology employing camelid-based nanobody is promising in this regard (393). Secondly, these novel radiotracers are not readily available due to the production complexity and cost compared to [^18^F]FDG (394, 395). Future research into an improved methodology for the economical and rapid production of these novel tracers is imperative to bring these into clinical practice. Finally, although theoretically the novel radiotracers mentioned in this review may be useful for the imaging of GCA and PMR, clinical trials are needed to evaluate and confirm their utility in the diagnosis and monitoring of GCA and PMR.

## Summary and Conclusion

Due to progress in our understanding of the immunopathology of GCA/PMR and the development of novel, highly specific tracers, direct imaging of immune cells/mediators by PET is now within reach. Such novel PET imaging strategies targeting a specific subset of inflammatory cells and activation markers of resident cells could be valuable diagnostic tools in GCA/PMR. Furthermore, direct imaging of infiltrating immune cells and inflammatory mediators might be useful for the treatment monitoring of GCA and PMR patients. Eventually, these novel radiotracers may also hold promise for disease stratification in GCA/PMR, since these tracers could help to select patients that may benefit from particular treatment regimens. The majority of these novel radiotracers are still mainly used as research tools in academic centers. Efforts are needed to evaluate these radiotracers in larger clinical trials to validate their utility in clinical practice. The introduction and implementation of such novel tracers will require close collaboration between patients, clinicians (e.g., rheumatologists, internists), nuclear medicine specialists and immunologists.

## Author Contributions

KG: conceptualization, conducted a review of the literature, and writing—review and editing. MS, PN, RS, and PH: writing—review and editing. EB: conceptualization and writing—review and editing. WJ: conceptualization, conducted a review of the literature, writing—original draft preparation, and writing—review and editing. All authors provide approval for publication of the content and agreed to be accountable for all aspects of the work.

## Funding

This review was supported by the Immune-Image consortium. The Immune-Image project receives funding from the Innovative Medicines Initiative 2 Joint Undertaking (JU) under grant agreement No 831514 (Immune-Image). The JU receives support from the European Union's Horizon 2020 research and innovation programme and EFPIA. This review was also supported by a research grant from FOREUM Foundation for Research in Rheumatology.

## Conflict of Interest

KG has received a speaker fee from Roche paid to the UMCG. EB has received consultancy and speaker fees from Roche paid to the UMCG. The remaining authors declare that the research was conducted in the absence of any commercial or financial relationships that could be construed as a potential conflict of interest.

## Publisher's Note

All claims expressed in this article are solely those of the authors and do not necessarily represent those of their affiliated organizations, or those of the publisher, the editors and the reviewers. Any product that may be evaluated in this article, or claim that may be made by its manufacturer, is not guaranteed or endorsed by the publisher.
